# Influence of Right Atrial Pressure on the Prognosis of Patients with Rheumatic Mitral Stenosis Undergoing Percutaneous Mitral Balloon Valvuloplasty

**DOI:** 10.3390/diagnostics14182079

**Published:** 2024-09-19

**Authors:** Daniella Cian Nazzetta, Larissa Christine Gomes de Sousa, Vitor Emer Egypto Rosa, Fernanda Castiglioni Tessari, Carlos M. Campos, Maria Antonieta Albanez Medeiros Lopes, Carlos Viana Poyares Jardim, Luís Gustavo Mapa, Layara Fernanda Vicente Pereira Lipari, Mariana Pezzute Lopes, João Ricardo Cordeiro Fernandes, Antonio de Santis, Lucas José Neves Tachotti Pires, Roney Orismar Sampaio, Flávio Tarasoutchi

**Affiliations:** Instituto do Coracao (InCor), Hospital das Clinicas HCFMUSP, Faculdade de Medicina, Universidade de Sao Paulo, Sao Paulo 05403-900, SP, Brazil; dani.nazzetta@gmail.com (D.C.N.); larissa.cg.sousa@gmail.com (L.C.G.d.S.); ftessari92@gmail.com (F.C.T.); carlosacampos1@gmail.com (C.M.C.); tietaalbanez@gmail.com (M.A.A.M.L.); carlos.jardim@hc.fm.usp.br (C.V.P.J.); luis_mapa_1@hotmail.com (L.G.M.); layaralipari@gmail.com (L.F.V.P.L.); mari.pezzutelopes@gmail.com (M.P.L.); jrcfernandes@hotmail.com (J.R.C.F.); antonio.santis@einstein.br (A.d.S.); lucasjtp@hotmail.com (L.J.N.T.P.); sampaioroney@yahoo.com.br (R.O.S.); tarasout@uol.com.br (F.T.)

**Keywords:** pulmonary hypertension, mitral stenosis, rheumatic fever, invasive pulmonary pressure, pulmonary vascular resistance

## Abstract

Background: Pulmonary hypertension (PH) often complicates mitral stenosis (MS). The prognostic impact of pulmonary vascular resistance (PVR) in MS patients remains unclear. Previous study has demonstrated the prognostic impact of right atrial pressure (RAP) in patients with primary PH. We aim to determine the prognostic impact of PVR and RAP in patients with rheumatic MS undergoing percutaneous mitral balloon valvuloplasty (PMBV). Methods: A total of 58 patients with symptomatic severe rheumatic MS who underwent PMBV between 2016 and 2020 were included. Patients were divided into two groups: PVR ≤ 2WU (N = 26) and PVR > 2WU (N = 32). The composite endpoint included death, reintervention or persistent NYHA functional class III-IV during follow-up. Results: The median age was 50 (42–60) years, with 82.8% being female. Median pulmonary artery systolic pressure (PASP) was 42 (35–50.5) mmHg. Patients with PVR ≤ 2WU had lower PASP on both echocardiogram and catheterization. The PMBV success rate was 75.9%. Multivariate analysis, adjusted for PVR, showed RAP as the only independent predictor of the composite endpoint (HR:1.507, 95% CI:1.015–2.237, *p* = 0.042). The optimal RAP cutoff was 9.5 mmHg (HR:3.481, 95% CI:1.041–11.641; *p* = 0.043). Conclusions: RAP was an independent predictor of adverse outcomes in patients with rheumatic MS undergoing PMBV, while PVR did not show prognostic significance. These findings suggest that the prognostic value of PVR may be lower than expected.

## 1. Introduction

Pulmonary hypertension is a frequent complication in patients with valvular heart disease, especially in those with mitral stenosis. In these patients, pulmonary artery systolic pressure (PASP) values ≥50 mmHg by echocardiogram at rest and PASP ≥ 60 mmHg at exercise are prognostic factors and, therefore, indicative for valve intervention [1,2,3,4]. Percutaneous mitral balloon valvuloplasty has been shown to be a safe and effective procedure for treating eligible rheumatic mitral stenosis, including patients with severe pulmonary hypertension [5,6,7].

The gold-standard method to evaluate pulmonary pressure is the hemodynamic assessment by catheterization, and a mean pulmonary artery pressure (mPAP) > 20 mmHg defines pulmonary hypertension [8]. However, isolated mPAP assessment fails to predict pulmonary vascular remodeling. It is crucial to evaluate of the pulmonary vascular resistance (PVR) and pulmonary arterial wedge pressure measures in order to distinguish pulmonary hypertension either due to pulmonary vascular disease or left heart disease [8]. Previous studies have shown conflicting results regarding the prognostic value of invasive pulmonary pressures measures in patients with left heart disease, and the literature is contradictory in defining whether these parameters indicate definitive or reversible pulmonary vascular disease [9,10,11,12]. Furthermore, a study conducted by Gilbert E. D’Alonzo et al. unveiled that hemodynamic measurements of the right chambers can have prognostic implications in these patients, and in this context, right atrial pressure (RAP) measurements appear to have impact on mortality [13].

The purpose of this study is to assess the impact of PVR, as a pre-capillary pulmonary hypertension surrogate, and RAP on the post-procedure outcomes of patients with rheumatic mitral stenosis undergoing percutaneous mitral balloon valvuloplasty.

## 2. Materials and Methods

### 2.1. Study Design

This is a unicentric, retrospective study, including 58 consecutive patients with symptomatic severe rheumatic mitral stenosis according to the current guidelines [1,2,3,14] who underwent percutaneous mitral balloon valvuloplasty between 2016 and 2020. Patients who did not have hemodynamic measurements evaluated during the procedure, pregnant women and emergency procedures were excluded. All patients underwent clinical and laboratory evaluation, electrocardiogram, pre-procedure transthoracic echocardiogram, pre-procedure and intraprocedural transesophageal echocardiogram and invasive pulmonary pressure measurements. They were divided according to the presence of pre-capillary pulmonary hypertension: PVR ≤ 2 woods unit (WU) (N = 26) and PVR > 2 WU (N = 32). Moreover, intra- and post-procedure complications were evaluated, in addition to mortality, hospitalization, New York Heart Association (NYHA) functional class and echocardiographic measurements within 30 days. The composite endpoint included death, reintervention and persistent NYHA functional class III-IV in the last follow-up contact.

### 2.2. Transthoracic and Transesophageal Doppler Echocardiographs

All transthoracic echocardiogram exams were analyzed in a central echocardiography laboratory using the same equipment (Vivid 9, GE Healthcare, Milwaukee, WI, USA or EPIQ 7, Koninklijke Philips N.V Amsterdam, Noord-Holland, The Netherlands). Severe mitral stenosis was defined in the presence of a mitral valve area ≤ 1.5 cm^2^ and/or a transmitral diastolic gradient ≥ 10 mmHg. The mitral valve area was calculated by planimetry, the Pressure Half Time (PHT) method, the Hatle formula (220/PHT) and/or the continuity equation, as appropriate. The gradient was obtained by the simplified Bernoulli equation (ΔPressure = 4 v^2^, where *v* is the transmitral velocity) [14,15]. The “Wilkins-Block” Score calculation was used for formal indication of the percutaneous mitral balloon valvuloplasty, and the score should have a value ≤8, and a maximum score of 2 for each calcification and subvalvular apparatus, as previous described [1,14,15]. Transesophageal echocardiogram was performed using the same equipment, immediately before and during percutaneous mitral balloon valvuloplasty. Patients with left atrial thrombus and moderate to severe mitral regurgitation were contraindicated for the procedure [15,16].

### 2.3. Percutaneous Mitral Balloon Valvuloplasty

The procedure is performed via a transfemoral venous approach with the patient under light sedation (RAAS (Richmond Agitation-Sedation Scale) − 2). After bolus administration of heparin, right heart catheterization including arteriography is performed and the septal puncture site is determined to the transseptal catheterization via a standard Brockenbrough procedure. A guidewire is placed into the left atrial, the Inoue balloon catheter is advanced across the interatrial septum and is inflated with contrast media guided by pressure measurements of the left chambers and by transesophageal echocardiogram. The balloon size was selected according to the patient’s weight or body surface area and by direct echocardiographic measurement of the mitral annular diameter [16,17]. The criteria for procedure success were at least one of the following, in addition to the absence of post-procedure moderate or severe mitral regurgitation: (1) increase in mitral valve area of >50% (or at least 0.5 cm^2^) or final mitral valve area ≥ 1.50 cm^2^; (2) mean gradient reduction from more than 10 to less than 5 mmHg; (3) transmitral gradient pressure reduction from an average of nearly 18 to 6 mmHg, with a small increase in cardiac output (average 20%) and double the calculated mitral valve area, from 1 to 2 cm^2^ [14,15,16,17,18,19].

### 2.4. Hemodynamic Measurements

Invasive pulmonary and intracardiac measurements were performed before and immediately after the procedure. Both venous and arterial punctures were made in order to enable the measurement of chamber pressures. A central venous puncture (most commonly internal jugular right or left) was used to access the right chambers using a Swan-Ganz catheter. An arterial puncture (radial or femoral artery) was used to access the left ventricle (bypassing aortic valve) and evaluate its pressures using a PigTail catheter. Through those measures, PVR ((mPAP—pulmonary arterial wedge pressure)/cardiac output) and the transpulmonary gradient, transmitral gradient and systemic vascular resistance were calculated. Cardiac output was determined using the Fick method or thermodilution [16,20].

### 2.5. Statistical Analysis

Continuous variables are shown as medians (interquartile range), while categorical variables as frequencies and percentages. Continuous variables were analyzed using the Mann–Whitney test and categorical variables using Fisher’s exact test or chi-square test, as appropriate. Cox regression analysis was used to assess predictors of the combined outcome (death, reoperation, new percutaneous mitral balloon valvuloplasty or long-term maintenance of NYHA functional class III/IV dyspnea). Variables with *p* < 0.05 in the univariate analysis were included in the multivariate model, which was adjusted for binary variable PVR > 2 Wood units (WU). Combined event-free survival was assessed using the Kaplan–Meier method and differences between groups were analyzed using the log-rank test. The Youden Index was used to define the best cut-off value for the variables predicting a composite outcome. A *p* value < 0.05 was considered statistically significant. All analyses were conducted using the SPSS statistical package, version 20 (IBM, Armonk, NY, USA).

## 3. Results

### 3.1. Patient Characteristics

A total of 58 patients were included; their clinical characteristics and laboratory data are summarized in Table 1. The median age was 50 (42–60) years, there was a female predominance (82.8%) and 56.9% had functional class II by the NYHA classification. The median EuroSCORE II was 1.3 (1.0–2.5) % and there was a high prevalence of comorbidities such as hypertension (55.2%), previous valvular intervention (22.4%), diabetes (20.7%) and atrial fibrillation (31%). There were no differences regarding baseline characteristics between the groups with pre-capillary pulmonary hypertension (PVR > 2 WU) and without pre-capillary pulmonary hypertension (PVR ≤ 2 WU). The baseline characteristics according to invasive right atrial pressure are shown in Supplemental Table S1.

### 3.2. Pre-Procedure Transthoracic Echocardiographic and Hemodynamic Data

The pre-procedure echocardiographic and hemodynamic data are summarized in Table 1 and Table 2. The median mitral valve area was 1.2 (0.9–1.3) cm^2^, the mean transmitral gradient was 8 (6–12) mmHg and the PASP was 42 (35–50.5) mmHg. There were no differences between the groups regarding pre-procedure echocardiographic parameters, except for PASP, which was lower in the PVR ≤ 2 WU group (37 (30–45) vs. 45 (40–56) mmhg, *p* = 0.011). Regarding the pre-procedure hemodynamic measurements, the right atrial pressure (RAP) was 8 (6–10) mmHg, without a difference between the groups. However, the PVR ≤ 2 WU group had lower PASP (33 (27,28) vs. 37 (36–52) mmHg, *p* = 0.007), mPAP (20 (15–22) vs. 29 (23–33) mmHg, *p* = 0.003), mean transpulmonary gradient (mTPG) (5 (3–6) vs. 10 (8–15) mmHg, *p* < 0.001), transpulmonary diastolic gradient (1 (0–2) vs. 5 (2–7) mmHg, *p* < 0.001), and PVR (1.3 (0.9–1.6) vs. 3.2 (2.4–4.3) mmHg/min, *p* < 0.001) compared to PVR > 2 WU group. The pre-procedure echocardiographic and hemodynamic data according to invasive right atrial pressure are shown in Supplemental Tables S2 and S3. Echocardiographic PASP was higher in patients with RAP ≥ 9.5 mmHg (46 (38–55) vs. 38.5 (32.2–45.5) mmHg, *p* = 0.035).

### 3.3. Procedure Data

The procedure data are summarized in Table 2. The percutaneous mitral balloon valvuloplasty success rate was 75.9%, with no difference between groups. In most cases, only one dilation was required (35, 71.4%). Ten patients (20.4%) underwent two dilations, whereas the remaining patients needed three or more dilations, with no difference between the groups. Conversion to open surgery was rare and occurred only in one patient from each group. The procedure data according to invasive right atrial pressure are shown in Supplemental Table S4.

### 3.4. Post-Procedure Echocardiographic and Hemodynamic Data

The echocardiographic and hemodynamic characteristics after the procedure are shown in Supplemental Tables S5 and S6. The PVR ≤ 2 WU group had lower mPAP (18 (15–23) vs. 23 (20–30) mmHg, *p* = 0.023), transpulmonary diastolic gradient (2 (0–3) vs. 4 (2–5) mmHg, *p* = 0.006) and PVR (1.42 (1.15–1.93) vs. 2.84 (2.24–3.35) mmHg/min, *p* < 0.001) compared to the PVR > 2 WU group. Considering the differences between the pre- and post-procedure values, the PVR ≤ 2 WU group had lower mTPG (−2.0 (−3.0–1.2) vs. 2.0 (−2.0–4.5) mmHg, *p* = 0.014) and PVR (−0.27 (−0.86–0.16) vs. 0.37 (−0.40–1.82) mmHg/min, *p* = 0.004) compared to the PVR > 2 WU group. However, changes in the diastolic transpulmonary gradient (*p* = 0.377) had no statistical significance between groups.

### 3.5. Outcomes

The post-procedure outcomes are summarized in Table 2. The median follow-up was 32.9 (20.2–43) months. During the follow-up, the need for reintervention (surgery or percutaneous mitral balloon valvuloplasty) happened in 6.9%, mortality was 1.7% and the composite endpoint occurred in 13 (22.4%) patients, with no difference between the groups.

**Figure 1:**
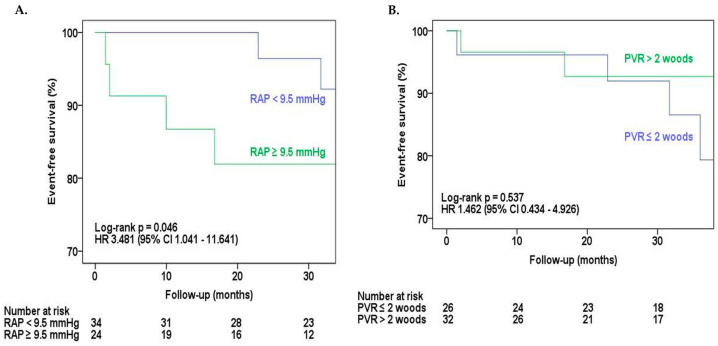
(**A**): Kaplan–Meier curve of the combined outcome-free survival (death, reoperation, new balloon-catheter mitral valvuloplasty or functional class III/IV NYHA on late follow-up) according to right atrial pressure on catheterization. RAP indicates right atrial pressure. (**B**): Kaplan–Meier curve of the combined outcome-free survival (death, reoperation, new balloon-catheter mitral valvuloplasty or functional class III/IV NYHA on late follow-up) according to pulmonary vascular resistance. PVR indicates pulmonary vascular resistance.

In the univariate analysis of combined outcome predictors (Table 3), four variables were associated with the composite endpoint: echocardiographic PASP (HR: 1.069, 95% CI 1.010–1.130, *p* = 0.021), RAP (HR: 1.267, 95% CI 1.028–1.562, *p* = 0.027), ∆ hemodynamic PASP (HR: 0.927, 95% CI 0.866–0.991, *p* = 0.026) and moderate or severe tricuspid regurgitation (HR: 6.318, 95% CI 1.734–23.023, *p* = 0.005). However, by multivariate analysis adjusted by PVR, RAP (HR: 1.507, 95% CI 1.015–2.237, *p* = 0.042) was the only independent predictor of the composite endpoint. A RAP value of 9.5 mmHg was the best cutoff to predict outcomes (HR 3.481, 95% CI 1.041–11.641; *p* = 0.043) (Figure 1A). A PVR greater than 2 WU was not a predictor of events, as well as the other hemodynamic variables evaluated (Figure 1B).

## 4. Discussion

The main findings of the present study were: 1—RAP measurement during catheterization was the only independent predictor of the combined outcomes in patients with severe mitral stenosis undergoing percutaneous mitral balloon valvuloplasty; 2—PVR was not a predictor of outcomes.

Pulmonary hypertension is associated with mitral stenosis and is considered an adverse prognostic indicator. Left heart disease represents the main cause of pulmonary hypertension, and it is responsible for about 50–85% of cases [21,22]. Weitsman et al. [23] showed that in patients with pulmonary hypertension and left heart disease, 51% presented valve dysfunction. The most common valve disease was mitral regurgitation, present in 66% of patients, and mitral stenosis was seen in 10% [23]. However, there are few studies regarding the prevalence of valve disease in patients with pulmonary hypertension; therefore, such data may be restricted to the analyzed region, with a low prevalence of rheumatic fever and thus mitral stenosis, unlike the present study. Pulmonary artery pressure values are usually obtained via echocardiography through PASP measurement, and it is considered severe when greater than 50 mmHg [1,2,3]. The hemodynamic pressure measurements by catheterization may vary according to patient volume status and heart rate; nevertheless, it is considered the gold standard for the assessment of intracardiac pressures and definition of pulmonary hypertension. Recently, the recommended value of mPAP to define pulmonary hypertension changed from ≥25 mmHg to >20 mmHg based on published data from three cohorts that demonstrated an increased mortality in individuals with an mPAP between 20 and 24 mmHg [8]. However, again, patients with left heart disease, including mitral stenosis, were underrepresented in these studies [24,25,26].

The mechanisms behind pulmonary hypertension in mitral stenosis are complex and not fully understood. Venocapillary hypertension from elevated left atrial pressures initially causes post-capillary PH, but if persistent, can induce pulmonary arterial vasoconstriction and remodeling, leading to a pre-capillary PH component defined by PVR > 2 WU [4,27,28]. These repercussions can affect right chambers, resulting in right ventricular dilatation and dysfunction, one of the key survival predictors in mitral stenosis. Right ventricular overload on electrocardiogram and elevated systolic pressures are associated with poor outcomes in mitral stenosis patients with pulmonary hypertension undergoing surgery, highlighting the importance of identifying right chamber involvement in patient management [29].

Studies regarding the prognostic value of invasive pulmonary pressures measures in patients with left heart disease are lacking and present conflicting results [8,10,11,30]. Tatsuro Ibe et. al. [12] demonstrated that pulmonary vascular disease classified by the diastolic pulmonary vascular pressure gradient in patients with pulmonary hypertension and left heart disease was associated with poor clinical outcomes when compared to those classified by the transpulmonary pressure gradient. Gerges et al. [10] also demonstrated that in patients with pulmonary hypertension due to left heart disease, an elevated diastolic pulmonary vascular pressure gradient can identify those who have associated pulmonary vascular remodeling. On the other hand, Robert Dragu et al. [11] demonstrated that PVR alone, but not diastolic pulmonary pressure, predicts worse outcomes in patients with pre and post-capillary pulmonary hypertension. It is important to emphasize that all these studies were retrospective and did not differentiate patients who underwent mitral valve procedure. Besides, no studies have specifically tested these parameters and thresholds in patients with severe mitral stenosis. In this context, patients with severe mitral stenosis may present a different pulmonary hypertension pattern for some reasons: (1) pulmonary hypertension is frequently present in mitral stenosis patients due to the high left atrial filling pressures; (2) as a pulmonary hypertension post-capillary component is always present, intervention usually improves pulmonary hypertension somehow [5]. In addition to outcomes, patients with PVR > 2.0 WU had similar clinical, laboratorial and echocardiographic characteristics compared to those with PVR ≤ 2.0 WU, except for PASP. Thus, the pulmonary pressure parameters could overestimate the impact of mitral stenosis on the pulmonary vasculature, minimizing the prognostic impact of PVR in this population.

In this way, the present study demonstrated that the PVR in patients with rheumatic mitral stenosis may be an overestimated measure with insufficient prognostic impact in this context. On the other hand, RAP measurement, as previously demonstrated, may be considered a prognostic marker [13]. Patients with RAP ≥ 9.5 mmHg could be in a more advanced stage of the pulmonary hypertension progression, and more attention should be given to the repercussions on the right chambers, despite invasive pulmonary pressure measurements [4]. Furthermore, RAP evaluation is easier, since Swan-Ganz catheter analysis would not be necessary and a central venous access would be enough to assess these measurements. It is also important to emphasize that none of these parameters are enough to contraindicate a mitral procedure in this group of patients. Until now, the prognostic parameters should only serve to indicate intervention and to plan specific postoperative care. Further prospective studies are needed to further evaluate the prognostic impact of RAP and PVR in mitral stenosis patients.

The main limitations of this study are the retrospective design and relatively small sample size that may have limited the power of the study. Also, hemodynamic measurements may vary according to the patient volume status and heart rate; however, it is considered the gold standard for defining pulmonary hypertension. Besides, patients eligible for percutaneous mitral balloon valvuloplasty are usually younger, in a less severe stage of the disease and have fewer comorbidities when compared to patients undergoing surgery, limiting the generalizability of the results. In addition, only rheumatic mitral stenosis patients were included and the results may not be generalizable to other populations or settings. Furthermore, RAP is a modifiable measure according to the patient’s volume status; however, all patients were already optimized on clinical therapy at the time of measurement.

## 5. Conclusions

In patients with severe mitral stenosis undergoing percutaneous mitral balloon valvuloplasty, the right atrial pressure measurement during catheterization (especially when above 9.5 mmHg) was an independent predictor of the combined outcome of death, reintervention and dyspnea functional class 3 or 4 at follow-up. Pre-capillary pulmonary hypertension was not a predictor of outcomes and PVR > 2.0 WU may overestimate the impact of mitral stenosis on the pulmonary vasculature.

## Figures and Tables

**Table 1:** Baseline characteristics and pre-procedure transthoracic echocardiography data of the overall population and according to pulmonary vascular resistance.

	Total (N = 58)	PVR ≤ 2 WU (N = 26)	PVR > 2 WU (N = 32)	*p*
Clinical Data				
Age, years	50.5 (42.0–60.5)	54.0 (43.0–62.5)	48.0 (40.2–59.5)	0.270
Body surface area, m^2^	1.70 (1.61–1.83)	1.69 (1.64–1.83)	1.72 (1.59–1.83)	0.851
Female sex	48 (82.8)	22 (84.6)	26 (81.3)	1.000
Diabetes mellitus	12 (20.7)	6 (23.1)	6 (18.8)	0.937
Hypertension	32 (55.2)	15 (57.7)	17 (53.1)	0.934
Previous valvular intervention	13 (22.4)	6 (23.1)	7 (21.9)	1.000
Atrial fibrillation	18 (31)	8 (30.8)	10 (31.3)	0.240
Coronary artery disease	1 (1.7)	0 (0)	1 (3.1)	1.000
Previous stroke or TIA	2 (3.4)	0 (0)	2 (6.3)	0.497
EuroSCORE II, %	1.3 (1.0–2.5)	1.3 (1.0–2.7)	1.1 (0.9–2.1)	0.219
Symptoms				
NYHA				0.140
II	33 (56.9)	18 (69.2)	15 (46.9)	
III	19 (32.8)	7 (26.9)	12 (37.5)	
IV	3 (5.2)	0 (0)	3 (9.4)	
Laboratory				
Hemoglobin, g/dL	13.6 (12.8–14.5)	13.5 (12.9–14.0)	13.8 (12.4–14.7)	0.814
Platelets/mm^3^	197,000 (167,500–229,500)	199,000 (175,000–224,500)	194,000 (163,250–233,250)	0.860
Creatinine, mg/dL	0.9 (0.8–1.0)	0.9 (0.8–1.0)	0.9 (0.8–1.0)	0.802
Creatinine clearance, ml/min/1.73 m^2^	76.5 (67.7–93.7)	75 (59–99)	79 (72–9)	0.352
Pre-procedure transthoracic echocardiography data				
LA volume, ml/m^2^	63 (53–74)	63 (47–72)	65 (53–78)	0.705
Interventricular septum, mm	9 (8–10)	9 (8–9.2)	9 (8–10)	0.777
LV posterior wall, mm	9 (8–9)	9 (8–9)	9 (8–9)	0.849
LV mass index, g/m^2^	88 (72–98)	91 (72–99)	84 (71–96)	0.521
LVDD, mm	48 (45–53)	50 (46–54)	47 (45–53)	0.219
LVSD, mm	32 (29–35)	32 (29–37)	31 (29–34)	0.541
LV diastolic, ml	108 (93–135)	118 (97–142)	102 (91–135)	0.164
LV systolic volume, ml	41 (32–51)	41 (32–58)	39 (32–47)	0.414
LVEF, %	62 (60–66)	63 (57–67)	62 (−60–65)	0.695
Mitral valve area, cm^2^	1.2 (0.9–1.3)	1.2 (1.0–1.3)	1.1 (0.9–1.2)	0.193
Maximum transmitral gradient, mmHg	18 (13–23)	14 (12–21)	19 (14–25)	0.129
Mean transmitral gradient, mmHg	8 (6–12)	7.5 (6–11)	9 (7–12)	0.214
PASP, mmHg	42 (35–50)	37 (30–45)	45 (40–56)	**0.011**
Wilkins score				0.252
5	1 (1.8)	0 (0)	1 (3.3)	
6	4 (7.3)	3 (12)	1 (3.3)	
7	23 (41.8)	12 (48)	11 (36.7)	
8	21 (38.2)	9 (36)	12 (40)	
9	6 (10.9)	1 (4)	5 (16.7)	
Moderate or severe tricuspid regurgitation	14 (24.6)	8 (30.8)	6 (19.4)	0.491

Values are median (interquartile range) or n (%). TIA indicates transient ischemic attack; NYHA, New York Heart Association; LA, left atrial; LV, left ventricular; LVEF, left ventricular ejection fraction; PASP, pulmonary artery systolic pressure; PVR, pulmonary vascular resistance; WU, Wood units. Bold values denote statistical significance.

**Table 2:** Pre-procedure and procedure data and outcomes of the overall population and according to pulmonary vascular resistance.

	Total (N = 58)	PVR ≤ 2 WU (N = 26)	PVR > 2 WU (N = 32)	*p*
Pre-procedure hemodynamic data				
Right atrial pressure, mmHg	8 (6–10)	7 (5–10)	9 (7–10)	0.067
Right ventricle systolic pressure, mmHg	36 (31–42)	35 (27–40)	37 (33–53)	0.025
Right ventricle diastolic pressure, mmHg	8 (6–10)	8 (6–10)	9 (6–10)	0.659
Pulmonary artery systolic pressure, mmHg	36 (31–42)	33 (27–38)	37 (33–52)	0.007
Pulmonary artery diastolic pressure, mmHg	20 (15–25)	20 (15–22)	22 (16–33)	0.030
Mean pulmonary artery pressure, mmHg	26 (21–31)	20 (15–22)	29 (23–33)	0.003
Pulmonary capillary pressure, mmHg	19 (15–22)	19 (16–22)	19 (14–22)	0.950
Mean transpulmonary gradient, mmHg	8 (5–11)	5 (3–6)	10 (8–15)	<0.001
Transpulmonary diastolic gradient, mmHg	3 (1–5)	1 (0–2)	5 (2–7)	<0.001
Transmitral gradient, mmHg	10 (6–13)	10 (6–11)	10 (7–14)	0.560
Left atrial pressure, mmHg	18 (15–22)	20 (16–22)	18 (15–23)	0.705
Left ventricle systolic pressure, mmHg	110 (100–120)	110 (100–120)	107 (93–122)	0.902
Left ventricle diastolic pressure, mmHg	10 (7–12)	8 (6–11)	10 (7–12)	0.373
Cardiac output, ml/min	3.9 (3.0–4.6)	4.0 (3.6–5.2)	3.4 (2.8–4.0)	0.003
Pulmonary vascular resistance, WU	2.1 (1.5–3.4)	1.3 (0.9–1.6)	3.2 (2.4–4.3)	<0.001
Procedure data				
Procedure success	44 (75.9)	20 (76.9)	24 (77.4)	1.000
Number of dilations, %				0.111
1	35 (71.4)	19 (82.6)	16 (61.5)	
2	10 (20.4)	4 (17.4)	6 (23.1)	
3	3 (6.1)	0 (0)	3 (11.5)	
4	1 (2)	0 (0)	1 (3.8)	
Outcomes				
Reintervention, %	4 (6.9)	2 (7.7)	2 (6.3)	1.000
Conversion to open surgery, %	1 (1.7)	1 (3.8)	0 (0)	0.448
30-day mortality	1 (1.7)	1 (3.8)	0 (0)	0.448
NYHA functional class in late follow-up				0.712
2	20 (35.1)	9 (36)	11 (34.4)	
3	8 (14)	3 (12)	5 (15.6)	
4	1 (1.8)	0 (0)	1 (3.1)	
NYHA functional class 3 or 4 in the late follow-up, n (%)	9 (15.5)	3 (11.5)	6 (18.8)	0.495
Composite endpoint	13 (22.4)	5 (19.2)	8 (25)	0.836

Values are median (interquartile range) or n (%). PVR indicates pulmonary vascular resistance; WU, Wood units; NYHA, New York Heart Association.

**Table 3:** Univariate and multivariate analysis of predictors of combined outcome (death, reintervention, maintenance of functional class 3 or 4 at late follow-up) adjusted for pulmonary vascular resistance.

	Univariate Analysis				Multivariate Analysis			
	HR	CI 95.0%		*p*	HR	CI 95.0%		*p*
		Inferior	Superior			Inferior	Superior	
PASP, mmHg	1.069	1.01	1.13	0.021	1.082	0.982	1.191	0.110
Right atrial pressure, mmHg	1.267	1.028	1.562	0.027	1.507	1.015	2.237	**0.042**
Δ pulmonary artery systolic pressure, mmHg	0.927	0.866	0.991	0.026	1.023	0.914	1.145	0.694
Moderate or severe tricuspid regurgitation	6.318	1.734	23.023	0.005	2.002	0.234	17.114	0.526
Pulmonary vascular resistance > 2.0 WU	1.462	0.434	4.926	0.540	0.916	0.124	6.776	0.932

Δ indicates the difference between pre and post procedure; PASP, pulmonary artery systolic pressure; WU, Wood units. Bold values denote statistical significance.

## Data Availability

Data are unavailable due to patient privacy and ethical restrictions.

## Supplementary Materials

The following supporting information can be downloaded at: https://www.mdpi.com/article/10.3390/diagnostics14182079/s1, Table S1: Baseline characteristics according to invasive right atrial pressure; Table S2: Pre-procedure transthoracic echocardiography data according to invasive right atrial pressure; Table S3: Pre-procedure hemodynamic data according to invasive right atrial pressure; Table S4: Procedure and post-procedure data and outcomes according to invasive right atrial pressure; Table S5: Post-procedure hemodynamic data of overall population and according to pulmonary vascular resistance; Table S6: Post-procedure in-hospital echocardiography data of overall population and according to pulmonary vascular resistance.
