# Supplementary material for: Influence of Right Atrial Pressure on the Prognosis of Patients with Rheumatic Mitral Stenosis Undergoing Percutaneous Mitral Balloon Valvuloplasty
Source: Diagnostics (Basel). 2024 Sep 19;14(18):2079. doi: 10.3390/diagnostics14182079 (PMC11431111; doi:10.3390/diagnostics14182079)
Supplement: Supplementary file 1 [file diagnostics-14-02079-s001.zip › diagnostics-3113974-supplementary.pdf]

Supplemental Table 1: Baseline characteristics according to invasive right atrial pressure

	RAP < 9.5mmHg (N=34)	RAP ≥ 9.5mmHg (N=24)	P
**Clinical Data**			
Age, years	45.5 (40.7-57.7)	55.5 (44-64.7)	0.070
Body surface area, m^2	1.69 (1.60-1.80)	1.74 (1.63-1.94)	0.230
Female sex	27 (79.4)	21 (87.5)	0.499
Diabetes Mellitus	8 (23.5)	4 (16.7)	0.744
Hypertension	16 (47.1)	16 (66.7)	0.226
Dyslipidemia	9 (26.5)	10 (41.7)	0.352
Smoking	7 (20.6)	6 (25)	0.938
Previous Valvular Intervention	7 (20.6)	6 (25)	0.938
Atrial fibrillation	9 (26.5)	9 (37.5)	0.544
Coronary artery disease	1 (2.9)	0 (0)	1.000
Previous stroke or TIA	2 (5.9)	0 (0)	0.506
EuroSCORE II, %	1.10 (0.95-2.29)	1.56 (1.13-2.96)	0.108
**Symptoms**			
NYHA			0.792
II	19 (55.9)	14 (58.3)	
III	12 (35.3)	7 (29.2)	
IV	2 (5.9)	1 (4.2)	
Angina	1 (2.9)	2 (8.3)	0.564
Syncope	2 (5.9)	0 (0)	0.506
**Medications**			
Aspirin	3 (8.8)	3 (12.5)	0.684
Warfarin	8 (23.5)	5 (20.8)	1.000
ACEI or ARB	5 (14.7)	5 (20.8)	0.726
Calcium channel blocker	3 (8.8)	3 (12.5)	0.684
Beta-blocker	25 (73.5)	20 (83.3)	0.574
Diuretics	20 (58.8)	18 (75)	0.319
Statin	5 (14.7)	4 (16.7)	1.000
Digoxin	5 (14.7)	8 (33.3)	0.175
Amiodarone	1 (2.9)	1 (4.2)	1.000
**Laboratory**			
Hemoglobin, g/dL	13.6 (12.7-14.2)	13.6 (12.9-14.7)	0.670
Platelets, /mm^3	196,000 (166,000-	202,000 (167,500-	0.698

	230,500)	228,500)	
Creatinine, mg/dL	0.9 (0.8-1.0)	0.9 (0.7-1.0)	0.492
Creatinine Clearance, ml/min/1.73 m^2	76 (66.2-92)	78 (72-111.2)	0.271

Values are median (interquartile range), or n (%). TIA indicates transient ischemic attack; NYHA, New York Heart Association; ACEI, angiotensin converting enzyme inhibitor; ARB, angiotensin receptor blocker; INR, international normalized ratio; RAP, right atrial pressure; WU, woods unit.

Supplemental Table 2: Pre-procedure transthoracic echocardiography data according to invasive right atrial pressure

	RAP < 9.5mmHg (N=34)	RAP ≥ 9.5mmHg (N=24)	P
Aortic sinus, mm	29.5 (27-31)	28 (26.2-30.7)	0.441
LA diameter, mm	51 (47-56)	50 (43.5-54.5)	0.512
LA volume, ml/m²	64 (46-78)	62 (55-71)	0.721
Interventricular septum, mm	8 (8-10)	9 (8.2-9.7)	0.215
LV posterior wall, mm	8.5 (8-9)	9 (8-9)	0.428
LV mass index, g/m²	89 (71.7-98.2)	84.5 (73-95.7)	0.705
LVDD, mm	48.5 (45-54)	48 (46-53)	0.924
LVSD, mm	31.5 (29.7-36.2)	32 (29.2-34)	0.905
LV diastolic, ml	108 (92-138)	108 (97-135)	0.848
LV systolic volume, ml	38 (33.5-54)	41 (32-47)	0.907
LVEF, %	62 (59.5-66)	64 (60-67)	0.579
Mitral valve area, cm²	1.2 (0.9-1.3)	1.1 (0.9-1.3)	0.732
Maximum transmitral gradient, mmHg	15 (13-23)	20 (15-25)	0.090
Mean transmitral gradient, mmHg	7 (6-11.2)	9 (6.2-12)	0.291
PASP, mmHg	38.5 (32.2-45.5)	46 (38-55)	**0.035**
Wilkins score			0.718
5	1 (3.1)	0 (0)	
6	3 (9.4)	1 (4.3)	
7	14 (43.8)	9 (39.1)	
8	11 (34.4)	10 (43.5)	
9	3 (9.4)	3 (13)	
Calcification			0.066
1	15 (46.9)	4 (17.4)	
2	16 (50)	18 (78.3)	
3	1 (3.1)	1 (4.3)	
Subvalvular apparatus			0.223
1	4 (12.5)	7 (30.4)	
2	26 (81.3)	14 (60.9)	
3	2 (6.3)	2 (8.7)	
Thickening			0.533
1	5 (15.6)	2 (8.7)	
2	24 (75)	20 (87)	

3	3 (9.4)	1 (4.3)	
Mobility			0.375
1	3 (9.4)	2 (8.7)	
2	28 (87.5)	18 (78.3)	
3	1 (3.1)	3 (13)	
Moderate or severe tricuspid regurgitation	5 (15.2)	9 (37.5)	0.104

Values are median (interquartile range), or n (%). LA indicates left atrium; LV, left ventricular; LVESD, left ventricular end-systolic diameter; LVESV, left ventricular end-systolic volume; LVEF, left ventricular ejection fraction; PASP, pulmonary artery systolic pressure; RAP, right atrial pressure. Bold values denote statistical significance.

Supplemental Table 3: Pre-procedure hemodynamic data according to invasive right atrial pressure

	RAP < 9.5mmHg (N=34)	RAP ≥ 9.5mmHg (N=24)	P
Right atrium pressure, mmHg	7 (5-8)	10 (10-12)	**<0.001**
Right ventricle systolic pressure, mmHg	35 (28-41.5)	38 (34-64.5)	**0.015**
Right ventricle diastolic pressure, mmHg	7 (5-8)	10 (10-13)	**<0.001**
Pulmonary artery systolic pressure, mmHg	34 (28-40.5)	38 (35-60)	**0.004**
Pulmonary artery diastolic pressure, mmHg	18 (15-22)	23 (18-34)	**0.002**
Mean pulmonary artery pressure, mmHg	24.5 (19.7-29)	29 (24-43)	**0.002**
Pulmonary capillary pressure, mmHg	17 (13.7-20)	21.5 (18-25)	**0.003**
Mean transpulmonary gradient, mmHg	6.5 (4-10)	9 (6-17)	**0.021**
Transpulmonary diastolic gradient, mmHg	2 (1-4)	5 (1.7-8.2)	**0.012**
Transmitral gradient, mmHg	10 (6.2-13)	9 (5.7-12)	0.645
Left atrium pressure, mmHg	17.5 (14.2-20.7)	22 (16-24)	0.069
Left ventricle systolic pressure, mmHg	103.5 (100-120)	116 (99-130)	0.354
Left ventricle diastolic pressure, mmHg	7 (6-9)	12 (10-15)	**<0.001**
Aorta systolic pressure, mmHg	105 (100-120)	100 (96.5-117)	0.910
Aorta diastolic pressure, mmHg	60 (60-66)	65 (60-75)	0.182
Cardiac output, ml/min	3.8 (3.1-4.6)	3.9 (2.8-4.4)	0.647
Pulmonary vascular resistance, mmHg/min	1.9 (1.2-2.5)	3.0 (1.5-4.8)	**0.016**

Values are median (interquartile range), RAP indicates right atrial pressure; WU, woods unit. Bold values denote statistical significance.

Supplemental Table 4: Procedure and post-procedure data and outcomes according to invasive right atrial pressure

	RAP < 9.5mmHg (N=34)	RAP ≥ 9.5mmHg (N=24)	P
**Procedure data**			
Procedure success	26 (76.5)	18 (78.3)	1.000
Number of dilations, %			0.250
1	25 (78.1)	10 (58.8)	
2	6 (18.8)	4 (23.5)	
3	1 (3.1)	2 (11.8)	
4	0 (0)	1 (5.9)	
**Outcomes**			
Reintervention, %	2 (5.9)	2 (8.3)	1.000
Conversion to open surgery, %	0 (0)	1 (4.2)	0.414
30-day mortality	1 (2.9)	0 (0)	1.000
NYHA functional class in late follow-up			0.078
2	13 (38.2)	7 (30.4)	
3	2 (5.9)	6 (26.1)	
4	0 (0)	1 (4.3)	
NYHA functional class 3 or 4 in the late follow-up, n (%)	2 (5.9)	7 (29.2)	**0.026**
Composite endpoint	4 (11.8)	9 (37.5)	**0.046**

Values are median (interquartile range), or n (%). NYHA indicates New York Heart Association; RAP, right atrial pressure;WU, woods unit.

Supplemental Table 5: Post-procedure hemodynamic data of overall population and according to pulmonary vascular resistance

	**Total (N=58)**	**PVR ≤ 2 WU (N=26)**	**PVR > 2 WU (N=32)**	**P**
Right atrium pressure, mmHg	8 (6 - 10)	7 (5 - 10)	8 (6 - 10)	0.147
Right ventricle systolic pressure, mmHg	32 (25 - 40)	30 (25 - 35)	35 (30 - 45)	0.053
Right ventricle diastolic pressure, mmHg	8 (6 - 10)	7.5 (4.7 - 10.0)	9 (7 - 10)	0.204
Pulmonary artery systolic pressure, mmHg	30 (25 - 40)	27 (25 - 36)	30 (2 - 45)	0.097
Pulmonary artery diastolic pressure, mmHg	15 (12 - 20)	15 (10 - 18)	17 (14 - 20)	0.092
Mean pulmonary artery pressure, mmHg	20 (17 - 27)	18 (15 - 23)	23 (20 - 30)	**0.023**
Pulmonary capillary pressure, mmHg	13 (11 - 15)	12 (10 - 15)	14 (10 - 16)	0.563
Mean transpulmonary gradient, mmHg	7 (5 - 10)	6 (4 - 7)	9 (6 - 13)	**0.002**
Transpulmonary diastolic gradient, mmHg	3 (1 - 5)	2 (0 - 3)	4 (2 - 5)	**0.006**
Transmitral gradient, mmHg	3 (2 - 5)	3 (2 - 5)	3 (2 - 5)	0.443
Left atrium pressure, mmHg	12 (10 -15)	12 (10 - 15)	13 (10 - 16)	0.529
Left ventricle systolic pressure, mmHg	108 (90 - 120)	100 (90 - 110)	109 (90 - 120)	0.403
Left ventricle diastolic pressure, mmHg	10 (8 - 13)	9 (7 - 12)	10 (9 - 13)	0.131
Aorta systolic pressure, mmHg	105 (90 - 120)	106 (99 - 120)	105 (90 - 120)	0.672
Aorta diastolic pressure, mmHg	60 (57 - 75)	63 (60 - 77)	60 (55 - 70)	0.700
Cardiac output, ml/min	3.8 (3.0 - 4.4)	4.0 (3.0 - 4.6)	3.8 (2.5 - 4.5)	0.410
Pulmonary vascular	2.09 (1.25 - 2.94)	1.42 (1.15 - 1.93)	2.84 (2.24 - 3.35)	**< 0.001**

resistance, mmHg/min				
Moderate or severe mitral regurgitation, %	3.0 (5.2)	2 (7.7)	1 (3.1)	0.582
Δ pulmonary artery systolic pressure, mmHg	5 (0 - 10)	3 (0 - 7)	8 (0 - 15)	**0.026**
Δ pulmonary artery diastolic pressure, mmHg	5 (2 - 7)	4.0 (0.7 - 5.0)	5 (2 - 10)	**0.050**
Δ mean pulmonary artery pressure, mmHg	4 (1 - 8)	3.0 (0.7 - 6.0)	7 (1 - 13)	**0.037**
Δ pulmonary capillary pressure, mmHg	5.0 (2.2 - 7.7)	5 (3 - 7.2)	4.5 (1.7 - 9.0)	0.954
Δ diastolic transpulmonary gradient, mmHg	0.0 (-2.0 - 2.0)	0.0 (-2.5 - 0.5)	0.0 (-2.0 - 2.5)	0.377
Δ mean transpulmonary gradient, mmHg	0.0 (-2.0 - 3.0)	-2.0 (-3.0 - 1.2)	2.0 (-2.0 - 4.5)	**0.014**
Δ pulmonary vascular resistance, mmHg/min	0.03 (-0.72 - 0.99)	-0.27 (-0.86 - 0.16)	0.37 (-0.40 - 1.82)	**0.004**

Values are median (interquartile range), or n (%). Δ indicates the difference between pre and post procedure; LA, left atrium; LV, left ventricular; LVESD, left ventricular end-systolic diameter; LVESV, left ventricular end-systolic volume; LVEF, left ventricular ejection fraction; PASP, pulmonary artery systolic pressure; PVR, pulmonary vascular resistance. Bold values denote statistical significance.

Supplemental Table 6: Post-procedure in-hospital echocardiography data of overall population and according to pulmonary vascular resistance

	Total (N=58)	PVR ≤ 2 WU (N=26)	PVR > 2 WU (N=32)	P
Aortic sinus, mm	29 (27 - 32)	29 (27 - 31)	29 (26 - 33)	0.949
LA diameter, mm	49 (46 - 54)	50 (45 - 53)	48 (45 - 54)	0.499
LA volume, ml/m²	50 (43 - 66)	51 (46 - 72)	49 (37 - 66)	0.427
Interventricular septum, mm	9 (8 - 10)	9 (8 - 9)	9 (8 - 10)	0.903
LV posterior wall, mm	8 (8 - 9)	8 (8 - 9)	9 (7 - 9)	0.384
LV mass index, g/m²	84 (70 - 103)	86 (72 - 106)	83 (69 - 99)	0.487
LVDD, mm	50 (46 - 53)	51 (46 - 54)	48 (45 - 52)	0.176
LVSD, mm	32 (30 - 35)	33 (30 - 35)	32 (29 - 35)	0.323
LV diastolic volume, ml	113 (92 - 130)	124 (90 - 135)	108 (93 - 124)	0.217
LV systolic volume, ml	41 (32 - 49)	44 (33 - 51)	39 (32 - 44)	0.299
LVEF, %	63 (60 - 65)	62 (60 - 65)	63 (60 - 65)	0.696
Mitral valve area, cm²	1.6 (1.4 - 1.6)	1.6 (1.5 - 1.6)	1.5 (1.3 - 1.6)	0.096
Maximum transmitral gradient, mmHg	10.0 (8.0 - 12.0)	10.0 (8.0 - 11.2)	10.0 (8.0 - 14.0)	0.538
Mean transmitral gradient, mmHg	4.0 (3.0 - 5.0)	4.0 (3.0 - 5.0)	4.0 (4.0 - 5.0)	0.131
PASP, mmHg	30.5 (23.2 - 37.2)	31.0 (23.0 - 35.0)	29.5 (24.2 - 38.0)	0.888
Moderate or severe mitral regurgitation, %	11 (20.8)	5 (22.7)	6 (29.4)	1.000
Moderate or severe tricuspid regurgitation, %	3 (5.7)	1 (4.5)	2 (6.5)	1.000

Values are median (interquartile range), or n (%). Δ indicates the difference between pre and post procedure; LA, left atrium; LV, left ventricular; LVESD, left ventricular end-systolic diameter; LVESV, left ventricular end-systolic volume; LVEF, left ventricular ejection fraction; PASP, pulmonary artery systolic pressure; PVR, pulmonary vascular resistance. Bold values denote statistical significance.
